# Difference in M2 Macrophage Infiltration Between Cell-Assisted Fat Graft and Biomaterial-Encapsulated Stem Cell-Assisted Fat Graft: An Experimental Animal Study

**DOI:** 10.3390/medicina62081516

**Published:** 2026-08-06

**Authors:** Phylicia Yan Yee Kam, Tzu-Hsun Tsai, Che-Wei Chang, Yo-Shen Chen

**Affiliations:** 1 Department of Human Appearance Center, Far Eastern Memorial Hospital, New Taipei City 220, Taiwan; phylicia.med.117@gmail.com; 2 Department of Ophthalmology, National Taiwan University Hospital, College of Medicine, National Taiwan University, Taipei 100, Taiwan; 3 Division of Plastic Surgery, Department of Surgery, Far Eastern Memorial Hospital, New Taipei City 220, Taiwan

**Keywords:** fat graft, biomaterial, cell-assisted, autologous fat transplantation, macrophage infiltration

## Abstract

*Background and Objectives*: Autologous fat transplantation using fat alone can lead to postoperative complications. Laminin–alginate beads have been investigated as carriers for adipose mesenchymal stem cells (ASCs), with the potential to modulate cell release kinetics, influence macrophage responses, and thereby affect the maintenance of graft volume. We hypothesized that encapsulating ASCs in laminin–alginate beads would allow gradual ASC release into the transplantation site and influence the macrophage-related microenvironment during fat graft maturation. *Materials and Methods*: ASCs were isolated from two female enhanced green fluorescent protein (eGFP) transgenic rats. For SC (ASC + fat) grafts, ASCs were freely mixed with 1 mL of mature adipose tissue. For bead-encapsulated cell (BEC) (laminin–alginate + ASC + fat) grafts, ASCs were first encapsulated in laminin–alginate beads and subsequently mixed with 1 mL of fat tissue. The fat graft was subcutaneously injected into the backs of the rats, which were subsequently euthanized at weeks 4, 6, and 8. The grafts were then harvested for histological and immunofluorescence analyses. *Results*: The histological analysis indicated that the number of eGFP-ASCs in the BEC grafts gradually increased between weeks 4 and 8, whereas that in the SC grafts decreased over the same period. The BEC grafts also exhibited a numerically higher degree of M2 macrophage infiltration. *Conclusions*: Laminin–alginate bead encapsulation was associated with prolonged persistence of eGFP-ASCs within the graft area. Although the macrophage-related findings were not statistically significant, they suggest a potential interaction between gradual ASC release and the local graft microenvironment.

## 1. Introduction

Fat grafting is widely used in plastic surgery because it offers high biocompatibility, easy access, and minimal damage to donor sites [1,2]. However, conventional fat grafts often exhibit unpredictable retention rates, with reported graft survival rates ranging between 24% and 79%, which may not be ideal [3,4]. Various approaches have been developed to improve the outcomes of fat grafting, including the use of cell-assisted fat grafts, which involve fat supplementation with adipose mesenchymal stem cells (ASCs) [5,6,7,8,9]. ASCs contribute to immunomodulation and paracrine signaling, increase proangiogenic factor secretion, promote epithelial cell differentiation, enhance neovascularization, and improve fat graft survival [10,11,12,13].

In previous studies [9,14], we incorporated biomaterials into fat grafting, wherein ASCs were encapsulated in porous laminin–alginate beads, which were subsequently incorporated into fat grafts prior to implantation. This biomaterial incorporation aimed to achieve the following goals: (1) tissue engineering of an artificial stem cell niche suitable for ASC attachment, (2) reducing the inflammatory response to ASCs, (3) facilitating tissue fluid circulation of nutrients after transplantation by increasing the space between adipocyte clusters, and (4) providing an ASC gradual-release system during the fat graft maturation period. This grafting method not only achieved a graft retention rate of 89% (which is similar to that for cell-assisted fat grafts reported previously, 63–90%) but also enhanced the final grafted mass via biomaterial addition to the initial graft volume [9,14].

Inflammatory cells play a crucial role in fat graft maturation. Upregulation of macrophage production, particularly M2 macrophage production, during fat grafting improves fat retention [15]. Therefore, manipulating macrophage polarization may be an approach for promoting neovascularization and increasing fat graft retention. However, the interactions between ASCs and macrophages within fat grafts, particularly in the presence of biomaterials, remain unclear.

The results of our previous studies can be attributed to interactions among laminin–alginate beads, ASCs, and fat tissues. In this study, we aimed to determine whether laminin–alginate beads can serve as a gradual-release system for ASCs, which in turn upregulate macrophage production during the fat graft maturation period.

## 2. Materials and Methods

### 2.1. Animals

ASCs were isolated from two 5–10-week-old female enhanced green fluorescent protein (eGFP) transgenic Sprague–Dawley (SD) rats (GEMMS-NLAC, Taipei, Taiwan). Eighteen autologous inguinal fat donors and graft recipients (6-week-old female SD rats [BioLASCO, Taipei, Taiwan]) were allocated in groups of three for each time point. This study was approved by the Institutional Animal Care and Use Committee of Far Eastern Memorial Hospital (IACUC-2018-FEMH-11).

### 2.2. eGFP-ASC Extraction

eGFP-ASCs were extracted from two eGFP SD rats, following a previously described method [16,17]. Briefly, inguinal fat was harvested, chopped, suspended in phosphate-buffered saline (PBS), and digested in type I collagenase (Gibco; Thermo Fisher, Waltham, MA, USA) for 4 h at 37 °C. The remaining tissue fragments were removed using a 70-µm strainer. The cell suspension was centrifuged for 5 min at 1000 rpm and washed twice with PBS. ASCs were then suspended in Dulbecco’s modified Eagle’s medium (Thermo Fisher Scientific, Waltham, MA, USA) supplemented with 1% penicillin–streptomycin and 10% fetal bovine serum. Non-adherent cells were removed every 3 days until confluence.

### 2.3. Preparation of Laminin–Alginate Beads with ASCs

**Figure 1:**
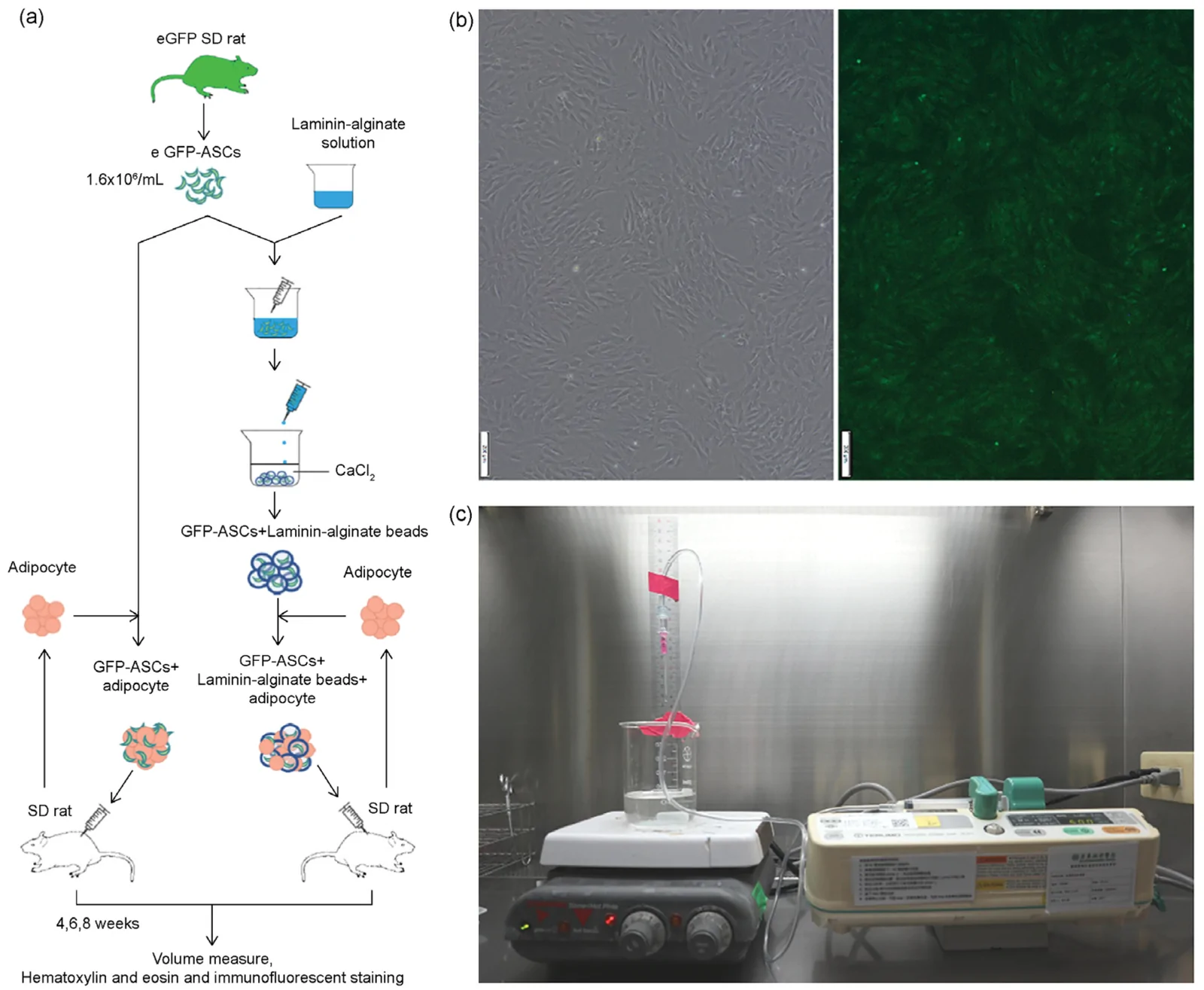
Animal fat graft model. (**a**) eGFP-ASCs extracted from donor-enhanced green fluorescent protein (eGFP) transgenic Sprague–Dawley (SD) rats. Some eGFP-ASCs were combined with the fat tissue of the recipient SD rats. Some eGFP-ASCs were added to the laminin–alginate solution and dropped into CaCl_2_ solution to form laminin–alginate beads with ASCs, which were then combined with the fat tissue of the recipient SD rats. (**b**) Microscopic morphology of eGFP-ASCs extracted from donor eGFP SD rats. Scale bars = 200 μm. (**c**) The laminin–alginate solution containing adipose mesenchymal stem cells in the syringe was dropped into CaCl_2_ solution in a beaker with a syringe pump to encapsulate the adipose mesenchymal stem cells in laminin–alginate beads.

ASC-containing laminin–alginate beads were prepared following a previously described procedure [14]. Briefly, 100 mL of 1.5% (*w*/*v*) alginate was mixed with 0.5 mL of 100 mM NaIO_4_ for 5 h. Thereafter, 0.5 mL of ethylene glycol was added to terminate the reaction. The solution was dialyzed (MWco: 6000–8000 Da) (Cellu-Sep T2; Dongil Biotech, Seoul, Republic of Korea) with distilled water for 3 days and freeze-dried to yield oxi-alginate powder. Laminin–alginate solution was prepared as follows: 1.5 g of oxi-alginate was dissolved in 100 mL of 20 mM 4-(2-hydroxyethyl)-1-piperazineethanesulfonic acid, and then 10 μg of laminin (Life Technologies; Thermo Fisher, Waltham, MA, USA) was added. The resulting solution was filtered through a sterile 0.22-μm membrane. Microspheres were quickly produced to ensure cell viability and prevent contamination. Laminin–alginate beads were prepared by mixing 1 mL of the laminin–alginate solution with 1.6 × 10^6^ eGFP-ASCs and dropping the resulting solution into 0.1 M CaCl_2_ at a stable speed through a 27G needle. The beads were transferred into sterile 0.9% NaCl solution and washed with gentle stirring for 1–2 min before use (Figure 1a–c).

### 2.4. Animal Model

We combined eGFP-ASCs from donor eGFP-SD rats with 1 mL of fat tissue from recipient SD rats to trace the ASCs and adipocytes after grafting. The inguinal fat was surgically removed after anesthetizing the rats and immersed in a 5-mL measuring cylinder filled with normal saline. The volume of fat was confirmed to be 1 mL by monitoring the increase in the liquid surface. The fat was used for subsequent analyses.

The rats were divided into two groups. Rats in the stem cell (SC) group were subcutaneously injected with mature adipocytes mixed with eGFP-ASCs. Rats in the bead-encapsulated cell (BEC) group were subcutaneously injected with adipocytes and eGFP-ASC laminin–alginate beads (Figure 1a). Each rat received two anatomically separate grafts, one on each dorsal side. Three animals per group were euthanized at each time point (4, 6, and 8 weeks), yielding six graft samples per group per time point (n = 6). This bilateral grafting approach enabled the efficient use of animals while maintaining the statistical validity.

### 2.5. Histological Analysis

The rats were euthanized at 4, 6, or 8 weeks after implantation, and the grafts were removed. All harvested samples were fixed with 4% paraformaldehyde in PBS, embedded in paraffin, sectioned at 4-μm thicknesses, and stained with hematoxylin and eosin (H&E) to evaluate the histological morphology.

### 2.6. Immunofluorescence Assays

The peripheries of lipid droplets in each sample were identified via immunofluorescence staining, and their distribution was assessed. Unstained sections measuring 4 µm in thickness were deparaffinized, subjected to antigen retrieval, blocked with 5% bovine serum albumin for 5 min, and incubated overnight with the following primary antibodies: rabbit anti-mouse perilipin-1 antibody (ab3526; Abcam, Cambridge, UK), rabbit anti-rat CD68 antibody (GTX37743; GeneTex, Irvine, CA, USA), rabbit anti-rat CD163 antibody (GTX35247; GeneTex), rabbit anti-rat NT5E antibody (DF6763; Affinity Biosciences, Cincinnati, OH, USA), or mouse anti-rat CD90.1 antibody (GTX76208; GeneTex). The sections were washed with PBS and then incubated for 1 h with the secondary antibodies Alexa Fluor 647-conjugated goat anti-rabbit IgG (ab150079, Abcam) or Cy™3 AffiniPure goat anti-rabbit IgG (AB_2338003; Jackson ImmunoResearch, West Grove, PA, USA). The samples were mounted using a mounting medium containing 4′,6-diamidino-2-phenylindole (a nuclei fluorescent stain) and observed using a tissue cytometer (TissueFAXS Spectra; TissueGnostics, Vienna, Austria) and imaging system (Metamorph) (Molecular Devices LLC, San Jose, CA, USA). For immunofluorescence quantification, regions of interest were selected within the graft area, excluding surrounding normal tissue, empty spaces, and staining artifacts. Fluorescence-positive signals were quantified using image-analysis software (METAMORPH; Molecular Devices, LLC, CA, USA, Versions 7.10). The CD163+/CD68+ value was calculated as a relative macrophage-related signal ratio.

### 2.7. Statistical Analysis

All values are presented as mean ± standard deviation (SD), and comparisons of parameters between the groups were performed using the unpaired Student’s *t*-test. Statistical significance was set at *p* ≤ 0.05. All statistical analyses were performed using SPSS version 25 (IBM Corp., Armonk, NY, USA).

## 3. Results

### 3.1. Higher eGFP-ASC Percentage in the BEC Group than in the SC Group

**Figure 2:**
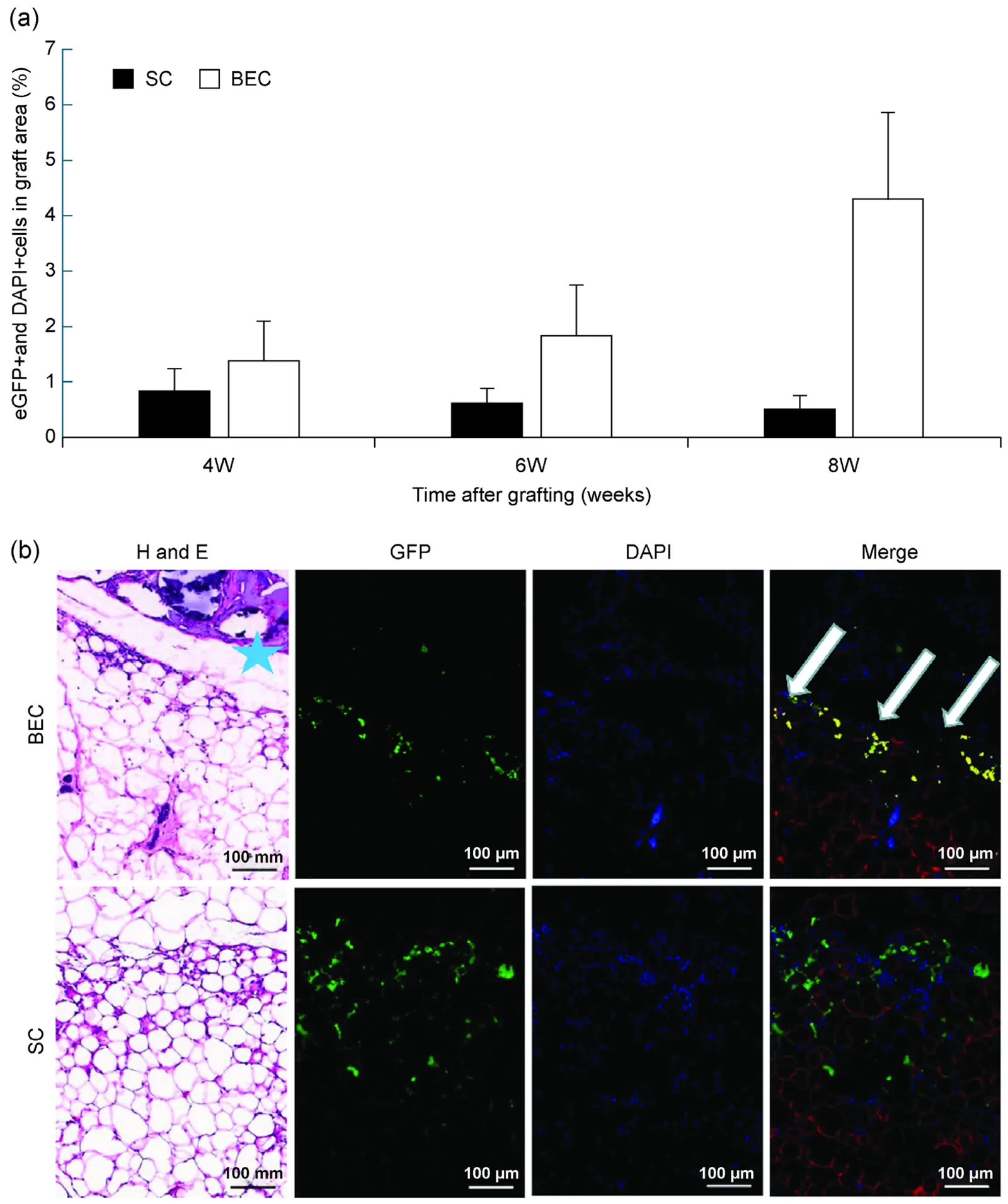
Analysis of the post-transplantation survival of eGFP-ASCs. (**a**) At 4, 6, and 8 weeks after grafting, the percentage of surviving eGFP+ and DAPI+ cells in the graft area of the BEC group was higher than that in the graft area of the SC group. Data are presented as mean ± SD. (**b**) Images of the graft material in the SC (below) and BEC (above) groups at postoperative week 8, obtained using tissue cytometry (TissueFAXS) and an imaging system (Metamorph). In the fluorescence images, green indicates GFP-positive donor-derived ASCs, blue indicates DAPI-stained nuclei, and red indicates perilipin-1-positive adipocytes. The merged images show the spatial relationship among eGFP-ASCs, nuclei, and adipocytes within the graft area. Residual laminin–alginate beads were observed in the BEC group and are indicated by the blue star. Arrows indicate GFP-positive ASCs near the bead-containing region in the BEC group. Magnification: ×200.

The percentages of eGFP+ and DAPI+ cells in the BEC group grafts increased gradually throughout the measurement period and were higher than those in the SC group grafts (SC: week 4, 0.87%; week 6, 0.65%; week 8, 0.52%; BEC: week 4, 1.40%; week 6, 1.85%; week 8, 4.32%) (Figure 2a,b). The P values at 6 and 8 weeks were 0.012 and 0.002, respectively, indicating a significant difference between the SC and BEC groups at these time points. At all time points (4, 6, and 8 weeks), the percentages of eGFP+ and DAPI+ cells in the graft area were consistently higher in the BEC group than in the SC group.

### 3.2. Higher Ratio of M2 Macrophages in the BEC Group

**Figure 3:**
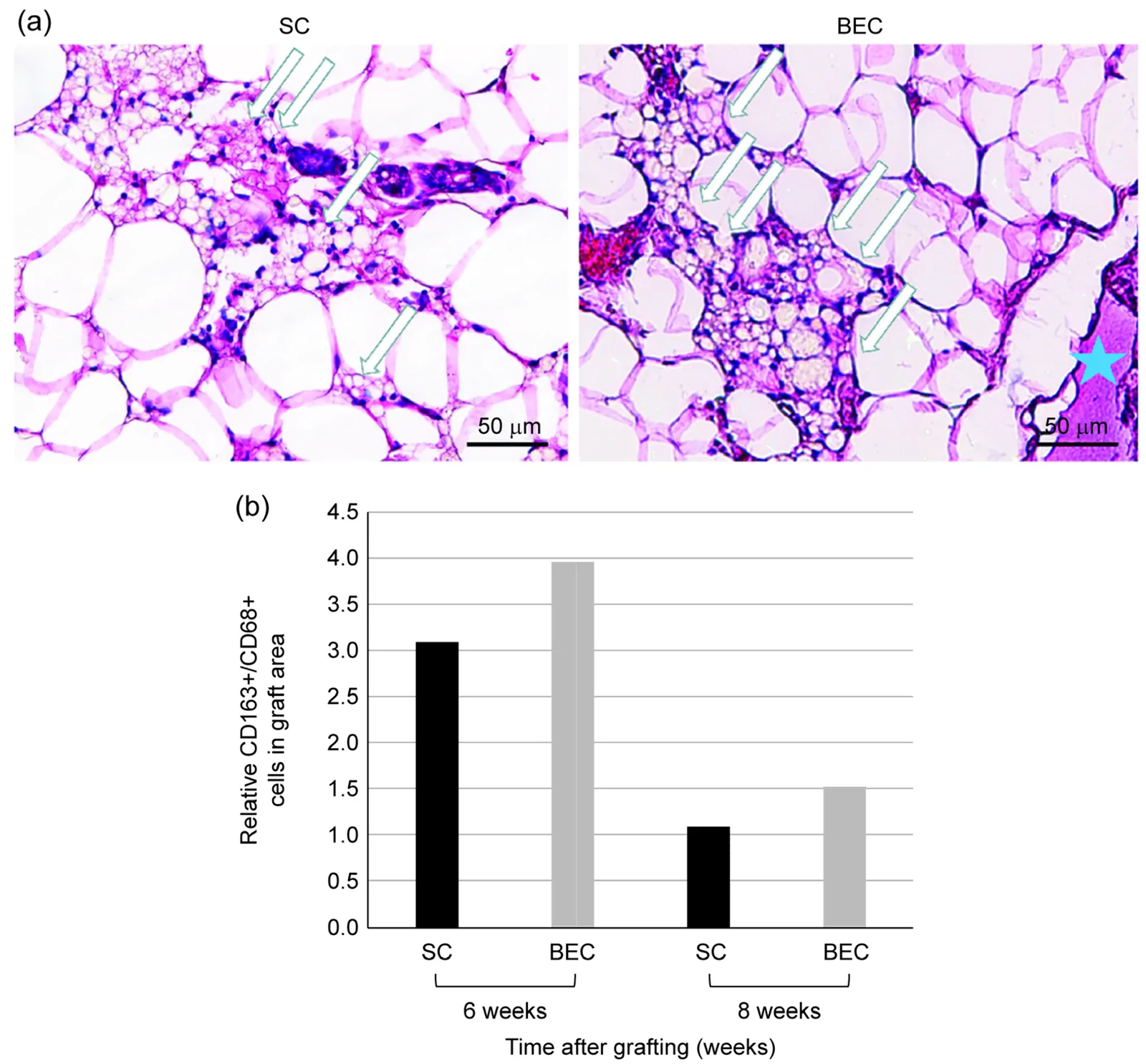
Analysis of post-transplantation M1 and M2 macrophage levels in the graft area. (**a**) Once the initial inflammation subsided and cell regeneration began, clusters of macrophages (arrows) were observed within the grafts in both groups (SC group, left; BEC group, right), under a microscope (Imager Z1; Carl Zeiss, Oberkochen, Germany) equipped with an imaging system (Nuance FX; PerkinElmer, Waltham, MA, USA). Residual laminin–alginate beads (blue stars) were observed in the BEC group. Magnification: ×400. (**b**) Relative CD163+/CD68+ cells in the graft area at postoperative weeks 6 and 8. Data are presented as mean ± SD.

We examined the grafts using H&E staining and found clusters of macrophages within the regenerated grafts in both SC and BEC groups (Figure 3a). At 6 weeks, the relative CD163+/CD68+ macrophage-related signal was numerically higher in the BEC group than in the SC group (SC group, 3.09; BEC group, 3.97) (Figure 3b). At 8 weeks, the relative CD163+/CD68+ signal remained numerically higher in the BEC group than in the SC group, although the values decreased in both groups (SC group, 1.10; BEC group, 1.49) (Figure 3b). The P values for the comparison between the groups at 6 and 8 weeks were 0.54 and 0.22, respectively, indicating that the differences between the SC and BEC groups at either 6 or 8 weeks were not significant. Additional statistical clarification, including the representative fluorescence quantification table and independent-samples *t*-test results, is provided in Supplementary Figure S1.

### 3.3. Increased Percentage of ASCs in the BEC Group

**Figure 4:**
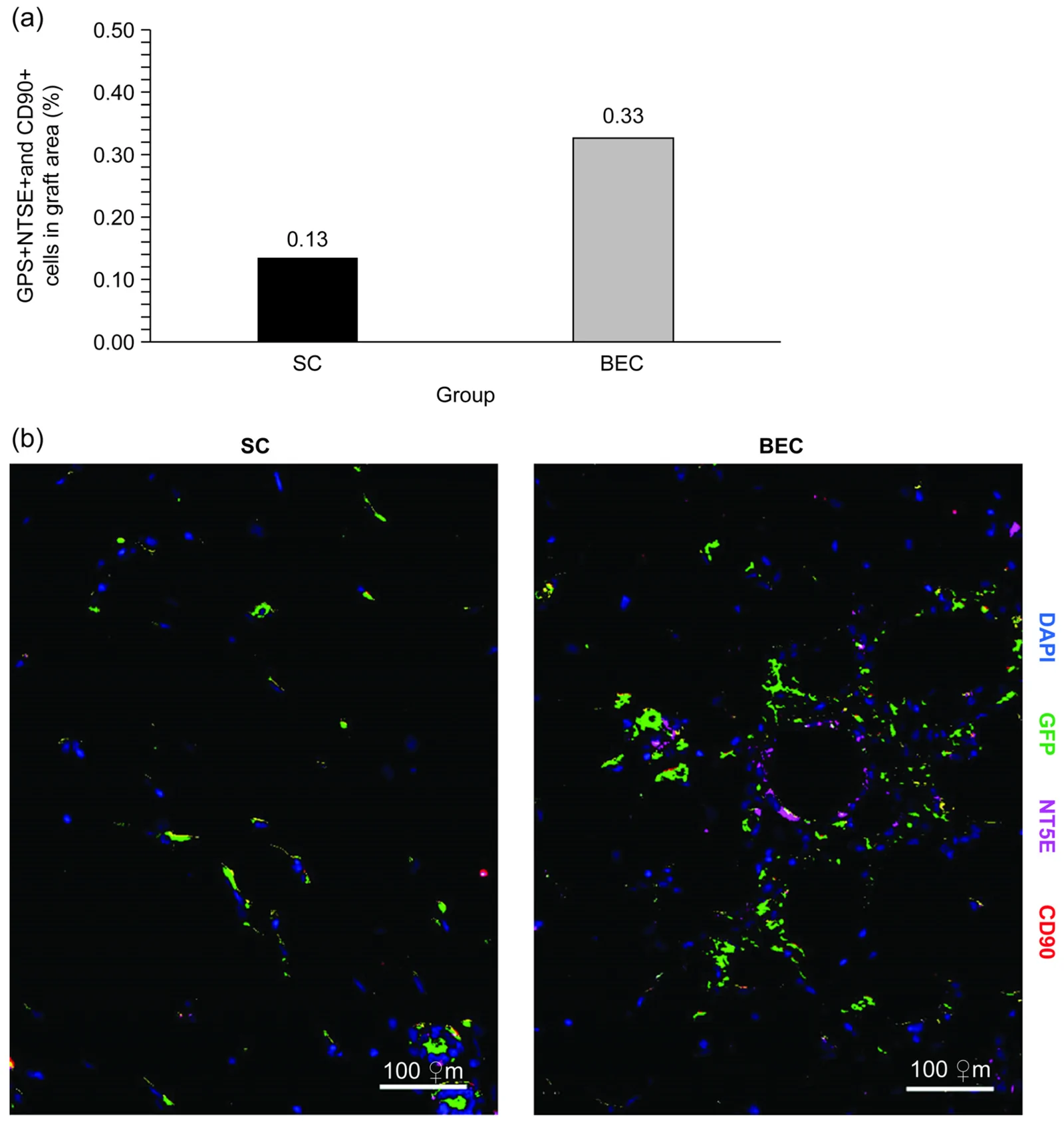
Analysis of post-transplantation ASC levels in the graft area. (**a**) Percentages of eGFP+, NT5E+, and CD90+ cells in the graft area 6 weeks after grafting. Fluorescence signals associated with ASCs in the graft were evaluated to determine ASC levels in the two groups. eGFP-ASCs in the BEC group were gradually released from the biomaterials, resulting in a higher percentage of ASCs in BEC grafts than in SC grafts. Data are presented as mean ± SD. (**b**) Images depicting staining of eGFP, NT5E, CD90, and DAPI in the SC (left) and BEC (right) groups were obtained using a microscope (Imager Z1; Carl Zeiss, Oberkochen, Germany) equipped with an imaging system (Nuance FX; PerkinElmer, MA, USA). Magnification: ×100.

The percentage of ASCs in the BEC group was higher than that in the SC group at 6 weeks after grafting (eGFP+, NT5E+, and CD90+ cells in the graft area: SC group, 0.13%; BEC group, 0.33%, *p* = 0.22) (Figure 4a,b).

## 4. Discussion

Cell-assisted fat grafts, developed to address the poor retention rates of conventional fat grafts [18,19,20], have numerous limitations despite achieving graft retention rates of 63–90% [5,6,7,8,9]. Accumulating evidence suggests that stem cells are located at sites of tissue damage or inflammation [21,22]. Thus, ASCs in cell-assisted fat grafts may respond to not only injured adipocytes within the graft but also the surrounding tissues injured while grafting, thereby reducing ASC availability for cellular regeneration. In the present study, the percentages of eGFP+ and DAPI+ cells in the SC group decreased gradually over 8 weeks (week 4, 0.87%; week 6, 0.65%; week 8, 0.52%) (Figure 2a). By contrast, the percentages of eGFP+ and DAPI+ cells in the BEC group increased over 8 weeks (week 4, 1.40%; week 6, 1.85%; week 8, 4.32%) (Figure 2b), indicating the gradual release of eGFP cells within the graft.

In our study, laminin–alginate was used as a scaffold and cell carrier. Alginate consists of polymerized (1–4)-linked β-d-mannuronate and its C-5 epimer α-l-guluronate. This copolymer can rapidly crosslink in the presence of divalent cations. Therefore, dropping the cell-containing alginate solution into a cationic bath enables instantaneous formation of porous beads that encapsulate the cells [23]. However, alginate is a negatively charged polysaccharide that has minimal interactions with mammalian cells. Therefore, we modified alginate with laminin, an extracellular matrix protein with terminal YGSIR and RGD peptide sequences known to enhance cell attachment and proliferation when incorporated into biomaterials [24]. Laminin–alginate exhibits greater biocompatibility than pure alginate beads when acting as a cell carrier [25]. The functions of ASCs are often dose-dependent. Therefore, the primary purpose of encapsulating ASCs was to enable their gradual migration to the graft rather than non-selective dispersion to injuries around it, thereby ensuring that ASCs are focused on the regeneration of fat cells [26]. Another potential benefit of encapsulating ASCs within biomaterials is that they mimic stem cell niches. Stem cell niches are microenvironments in which stem cells originally reside in vivo and receive stimuli that influence their behavior. ASCs isolated in vitro and expanded in 2D cultures gradually lose their functional properties [27,28,29]. Thus, the biomaterial may provide a niche-like 3D environment, protect and shield ASCs from the initial inflammatory insult, and retain their pluripotency and immunomodulatory properties. Here, some nutrients from the culture medium were encapsulated along with the ASCs during cell encapsulation, acting as a short-term source of nutritional support for stem cells.

A possible drawback of introducing biomaterials into fat grafts is the inevitable inflammatory response. However, the acute inflammatory response to biomaterials usually resolves in less than a week [30]. Initial neutrophil infiltration of the fat graft can be expected even without biomaterial introduction; however, the number of neutrophils decreases after 3 days, and macrophages begin to dominate and become the major inflammatory cell components within the graft [31]. Macrophages within the grafts were initially believed to be involved in free oil clearance and phagocytosis [32]. However, recent studies have shown that macrophages generate recruitment signals for stem cell graft infiltration [33,34]. Using macrophage models, Cai et al. [31] showed that the number of macrophages within a fat graft may be one of the factors that determine the density and number of newly formed host-derived blood vessels. They suggested that fat graft retention could be improved by macrophage enhancement. Thus, although the exact role of macrophages in the survival of fat grafts remains unclear, their presence indicates that the inflammation induced by biomaterials may not have an overwhelmingly negative effect on fat graft survival. Therefore, evaluating macrophage-related changes may help characterize the inflammatory and regenerative microenvironment during graft maturation.

Macrophage activation in response to signals varies with signal intensity. In general, M1 macrophages mediate inflammatory responses and express high levels of pro-inflammatory cytokines. The M2 macrophages produce anti-inflammatory cytokines that mediate tissue repair and inflammation [20]. Mesenchymal stem cells stimulate macrophages to produce anti-inflammatory and immunosuppressive cytokines, thereby inducing their polarization toward M2 macrophages. Using CD68+ and CD163+ markers, we evaluated fluorescent signals from all macrophages, including M2 macrophages, to estimate the inflammatory tendencies of the SC and BEC groups. The M2 macrophage levels after grafting were numerically higher in the BEC group than in the SC group at weeks 6 (SC group, 3.09; BEC group, 3.97) and 8 (SC group, 1.10; BEC group, 1.49). This difference may be due to the greater accessibility of ASCs within the BEC grafts, which, as suggested in a previous study, might lead to greater M2 polarization [16] (Figure 3a,b).

In a previous study, we attempted in vivo implantation of ASCs encapsulated in laminin–alginate without fat grafting. These implants reacted only to the biomaterial as a foreign body and displayed no signs of adipogenesis or vascular regeneration of ASCs [14]. Thus, in the absence of adipocytes, biomaterial-encapsulated ASCs may have limited capacity to undergo adipogenic differentiation in vivo and may not fully reproduce the microenvironment in which adipocytes secrete large amounts of cytokines and adipokines [35,36], and some of these secreted factors trigger adipogenic stem cell differentiation [37,38]. A mixture containing injury-associated growth factors in proportions identical to those found in exudate from injured adipose tissue can mitigate tissue hypoxia by increasing capillary density and ASC proliferation in ischemic and injured adipose tissue [39]. We speculate that similar exudates occur within the transferred fat grafts. Hence, the synergy among adipocytes, injury-induced exudates, and ASCs may facilitate enhanced regeneration and maturation of fat grafts in both cell-assisted and biomaterial-encapsulated stem cell grafts. Finally, once the fat grafts overcame the inflammatory response and began to regenerate, clusters of macrophages were observed within the regenerated tissue in both groups (Figure 3a). Macrophages can extend their periods of self-maintenance. In addition to their phagocytic functions, macrophages can adapt to their environment [40,41,42,43] and perform functions that are essential for organ homeostasis [44]. Macrophage cluster formation observed in the tissue samples in this study suggests that the initial inflammation had subsided and that the tissue had reached an equilibrium state. Nonetheless, further investigations are needed to gain a complete understanding of the functions and fates of these macrophage clusters in fat grafts, which still remain uncertain.

Additional processing with biomaterials in a certified laboratory during ASC preparation may be a clinically feasible option in the future and could be clinically applied for better volumetric augmentation or reconstruction. The present study was limited by the small number of animals, a relatively short follow-up period, and a lack of direct graft retention measurements due to the small graft size. As part of the experimental design, two grafts were placed in each recipient animal. Therefore, graft-level measurements from the same animal may not be fully biologically independent, as they could be influenced by shared host-related biological factors. Together with the small sample size, this limits the interpretation of group differences, and the findings should be considered exploratory. In addition, adipocytes alone and adipocytes mixed with laminin–alginate beads without ASCs were evaluated in our previous 7T MRI animal study, and these groups were not included in the present study; therefore, the independent contribution of the biomaterial itself cannot be fully distinguished from the effect of ASC encapsulation. Further studies with larger sample sizes, larger graft volumes, and longer follow-up periods are required to fully understand the potential benefits and contraindications of the biomaterial additive to the fat graft. Furthermore, definite volume evaluation using MRI should be considered in future studies.

**Figure 5:**
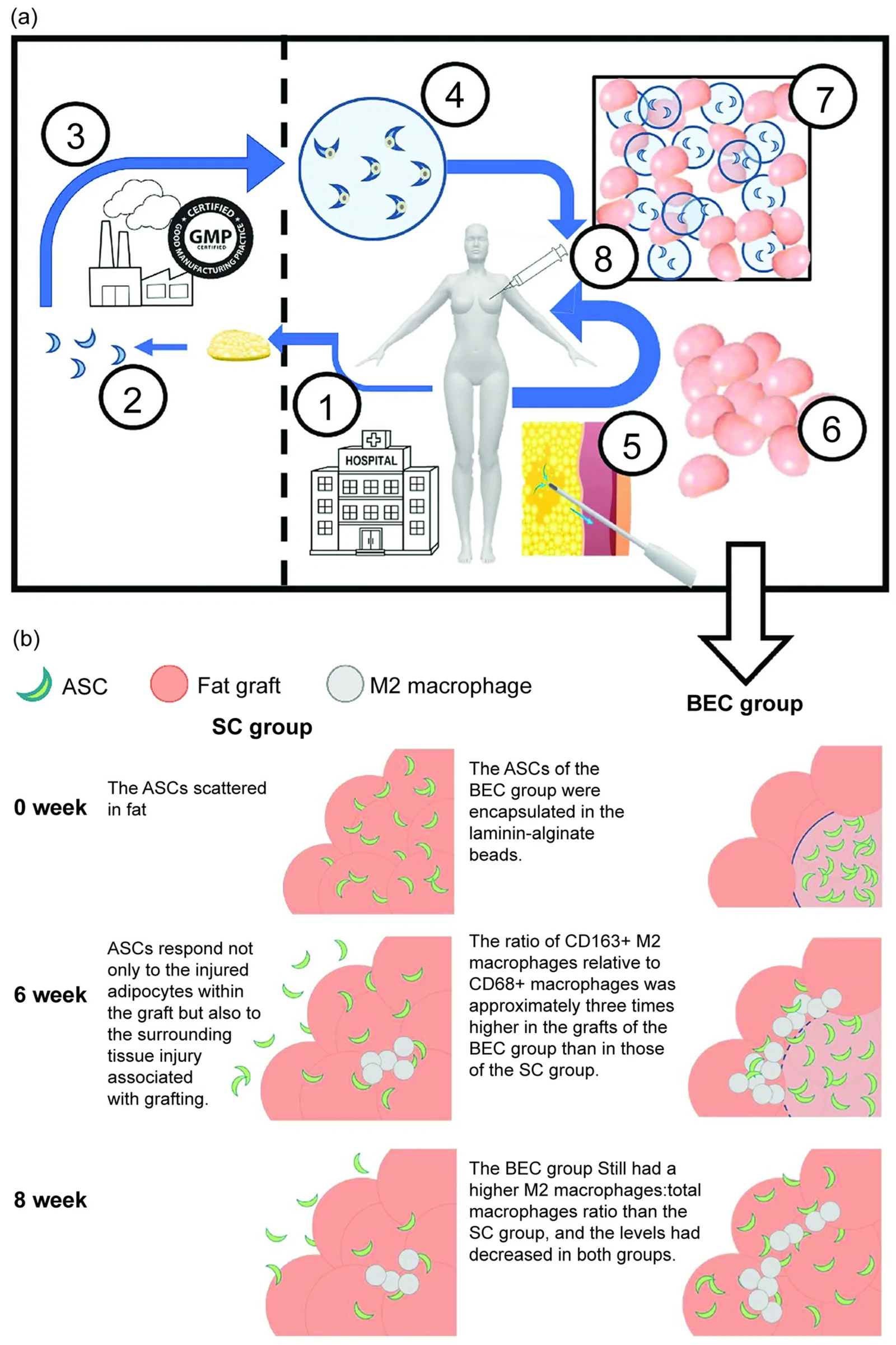
Schematic of the procedure for translating the results of this study to clinical settings in an autologous transplant procedure. (**a**) The procedure used in this study can be translated to a clinical scenario in an autologous manner. 1. Harvest 3 mL inguinal fat under local anesthesia. 2. Send the fat sample to a certified laboratory for ASC extraction. 3. Encapsulate ASCs within biomaterials in the laboratory. 4. Produce bead-encapsulated cells (BECs). 5. Perform liposuction. 6. Obtain adipocytes from lipoaspirate. 7. Mix adipocytes with BECs. 8. Perform soft tissue reconstruction or augmentation via the injection of the mixture. (**b**) The course of graft changes in SCs and BECs after transplantation.

A schematic for translating the procedure followed in this study into a clinical setting in an autologous manner is presented in Figure 5a,b. As ASC processing methods continue to advance, allogeneic ASC-based approaches may become feasible, although further evidence is needed before clinical translation.

## 5. Conclusions

Encapsulating ASCs within biomaterials is a feasible method for enhancing cell-assisted fat grafting. We consider this to be an exploratory animal study. Bead encapsulation was associated with prolonged persistence of eGFP-ASCs within the graft area, suggesting a possible gradual-release effect. The BEC group also showed a numerically higher, but non-significant, CD163+/CD68+ macrophage-related signal than the SC group. Based on these results, and due to the limited sample size, the use of biological materials for fat grafting warrants further investigation. A better understanding of fat graft maturation and complex intercellular and intracellular processes can help further fine-tune biomaterial constituents. This, in turn, improves ASC release and regulates the inflammatory response within the fat grafts.

## Data Availability

The original contributions presented in this study are included in the article. Further inquiries can be directed to the corresponding author.

## Supplementary Materials

The following supporting information can be downloaded at: https://www.mdpi.com/article/10.3390/medicina62081516/s1, Figure S1: Quantification and statistical analysis of CD163+/CD68+ macrophage-related signals.

## Abbreviations

The following abbreviations are used in this manuscript:

ASC	Adipose mesenchymal stem cell
BEC	Bead-encapsulated cell
eGFP	Enhanced green fluorescent protein
H&E	Hematoxylin and eosin
PBS	Phosphate-buffered saline
SC	Stem cell
SD	Sprague–Dawley

## References

[1] Cohen S.R., Hewett S., Ross L., Delaunay F., Goodacre A., Ramos C., Leong T., Saad A. (2017). Regenerative cells for facial surgery: Biofilling and biocontouring. Aesthetic Surg. J..

[2] Auclair E., Blondeel P., Del Vecchio D.A. (2013). Composite breast augmentation: Soft-tissue planning using implants and fat. Plast. Reconstr. Surg..

[3] Mineda K., Kuno S., Kato H., Kinoshita K., Doi K., Hashimoto I., Nakanishi H., Yoshimura K. (2014). Chronic inflammation and progressive calcification as a result of fat necrosis: The worst outcome in fat grafting. Plast. Reconstr. Surg..

[4] Kato H., Mineda K., Eto H., Doi K., Kuno S., Kinoshita K., Kanayama K., Yoshimura K. (2014). Degeneration, regeneration, and cicatrization after fat grafting: Dynamic total tissue remodeling during the first 3 months. Plast. Reconstr. Surg..

[5] Sterodimas A., de Faria J., Nicaretta B., Boriani F. (2011). Autologous fat transplantation versus adipose-derived stem cell-enriched lipografts: A study. Aesthetic Surg. J..

[6] Tiryaki T., Findikli N., Tiryaki D. (2011). Staged stem cell-enriched tissue (SET) injections for soft tissue augmentation in hostile recipient areas: A preliminary report. Aesthetic Plast. Surg..

[7] Castro-Govea Y., De La Garza-Pineda O., Lara-Arias J., Chacón-Martínez H., Mecott-Rivera G., Salazar-Lozano A., Valdes-Flores E. (2012). Cell-assisted lipotransfer for the treatment of Parry-Romberg syndrome. Arch. Plast. Surg..

[8] Tanikawa D.Y.S., Aguena M., Bueno D.F., Passos-Bueno M.R., Alonso N. (2013). Fat grafts supplemented with adipose-derived stromal cells in the rehabilitation of patients with craniofacial microsomia. Plast. Reconstr. Surg..

[9] Mizuno H., Tobita M., Uysal A.C. (2012). Concise review: Adipose-derived stem cells as a novel tool for future regenerative medicine. Stem Cells.

[10] Zuk P.A., Zhu M., Mizuno H., Huang J., Futrell J.W., Katz A.J., Benhaim P., Lorenz H.P., Hedrick M.H. (2001). Multilineage cells from human adipose tissue: Implications for cell-based therapies. Tissue Eng..

[11] Klinger F.M., Vinci V., Forcellini D., Caviggioli F. (2011). Basic science review on adipose tissue for clinicians. Plast. Reconstr. Surg..

[12] Gir P., Oni G., Brown S.A., Mojallal A., Rohrich R.J. (2012). Human adipose stem cells: Current clinical applications. Plast. Reconstr. Surg..

[13] Zhu M., Dong Z., Gao J., Liao Y., Xue J., Yuan Y., Liu L., Chang Q., Lu F. (2015). Adipocyte regeneration after free fat transplantation: Promotion by stromal vascular fraction cells. Cell Transplant..

[14] Chen Y.S., Hsueh Y.S., Chen Y.Y., Lo C.Y., Tai H.C., Lin F.H. (2017). Evaluation of a laminin-alginate biomaterial, adipocytes, and adipocyte-derived stem cells interaction in animal autologous fat grafting model using 7-Tesla magnetic resonance imaging. J. Mater. Sci. Mater. Med..

[15] Sica A., Mantovani A. (2012). Macrophage plasticity and polarization: In vivo veritas. J. Clin. Investig..

[16] Gao S., Mao F., Zhang B., Zhang L., Zhang X., Wang M., Yan Y., Yang T., Zhang J., Zhu W. (2014). Mouse bone marrow-derived mesenchymal stem cells induce macrophage M2 polarization through the nuclear factor-κB and signal transducer and activator of transcription 3 pathways. Exp. Biol. Med..

[17] Zuk P.A., Zhu M., Ashjian P., De Ugarte D.A., Huang J.I., Mizuno H., Alfonso Z.C., Fraser J.K., Benhaim P., Hedrick M.H. (2002). Human adipose tissue is a source of multipotent stem cells. Mol. Biol. Cell.

[18] Eto H., Kato H., Suga H., Aoi N., Doi K., Kuno S., Yoshimura K. (2012). The fate of adipocytes after nonvascularized fat grafting: Evidence of early death and replacement of adipocytes. Plast. Reconstr. Surg..

[19] Matsumoto D., Sato K., Gonda K., Takaki Y., Shigeura T., Sato T., Aiba-Kojima E., Iizuka F., Inoue K., Suga H. (2006). Cell-assisted lipotransfer: Supportive use of human adipose-derived cells for soft tissue augmentation with lipoinjection. Tissue Eng..

[20] Yoshimura K., Sato K., Aoi N., Kurita M., Hirohi T., Harii K. (2008). Cell-assisted lipotransfer for cosmetic breast augmentation: Supportive use of adipose-derived stem/stromal cells. Aesthetic Plast. Surg..

[21] Yagi H., Soto-Gutierrez A., Parekkadan B., Kitagawa Y., Tompkins R.G., Kobayashi N., Yarmush M.L. (2010). Mesenchymal stem cells: Mechanisms of immunomodulation and homing. Cell Transplant..

[22] Zhao Y., Zhang H. (2016). Update on the mechanisms of homing of adipose tissue-derived stem cells. Cytotherapy.

[23] Orive G., Hernández R.M., Gascón A.R., Calafiore R., Chang T.M., De Vos P., Hortelano G., Hunkeler D., Lacík I., Shapiro A.M. (2003). Cell encapsulation: Promise and progress. Nat. Med..

[24] Hohenester E., Tisi D., Talts J.F., Timpl R. (1999). The crystal structure of a laminin G-like module reveals the molecular basis of alpha-dystroglycan binding to laminins, perlecan, and agrin. Mol. Cell.

[25] Chen Y.S., Chen Y.Y., Hsueh Y.S., Tai H.C., Lin F.H. (2016). Modifying alginate with early embryonic extracellular matrix, laminin, and hyaluronic acid for adipose tissue engineering. J. Biomed. Mater. Res. Part A.

[26] Waldner M., Zhang W., James I.B., Allbright K., Havis E., Bliley J.M., Almadori A., Schweizer R., Plock J.A., Washington K.M. (2018). Characteristics and immunomodulating functions of adipose-derived and bone marrow-derived mesenchymal stem cells across defined human leukocyte antigen barriers. Front. Immunol..

[27] Jones D.L., Wagers A.J. (2008). No place like home: Anatomy and function of the stem cell niche. Nat. Rev. Mol. Cell Biol..

[28] Voog J., Jones D.L. (2010). Stem cells and the niche: A dynamic duo. Cell Stem Cell.

[29] Kaewsuwan S., Song S.Y., Kim J.H., Sung J.H. (2012). Mimicking the functional niche of adipose-derived stem cells for regenerative medicine. Expert Opin. Biol. Ther..

[30] Anderson J.M., Rodriguez A., Chang D.T. (2008). Foreign body reaction to biomaterials. Semin. Immunol..

[31] Cai J., Feng J., Liu K., Zhou S., Lu F. (2018). Early macrophage infiltration improves fat graft survival by inducing angiogenesis and hematopoietic stem cell recruitment. Plast. Reconstr. Surg..

[32] Ploeger D.T., van Putten S.M., Koerts J.A., van Luyn M.J., Harmsen M.C. (2012). Human macrophages primed with angiogenic factors show dynamic plasticity, irrespective of extracellular matrix components. Immunobiology.

[33] Lolmede K., Campana L., Vezzoli M., Bosurgi L., Tonlorenzi R., Clementi E., Bianchi M.E., Cossu G., Manfredi A.A., Brunelli S. (2009). Inflammatory and alternatively activated human macrophages attract vessel-associated stem cells, relying on separate HMGB1- and MMP-9-dependent pathways. J. Leukoc. Biol..

[34] Snyder R.J., Lantis J., Kirsner R.S., Shah V., Molyneaux M., Carter M.J. (2016). Macrophages: A review of their role in wound healing and their therapeutic use. Wound Repair. Regen..

[35] Coppack S.W., Pinkney J.H., Mohamed-Ali V. (1998). Leptin production in human adipose tissue. Proc. Nutr. Soc..

[36] Deng Y., Scherer P.E. (2010). Adipokines as novel biomarkers and regulators of the metabolic syndrome. Ann. N. Y. Acad. Sci..

[37] Stillaert F., Findlay M., Palmer J., Idrizi R., Cheang S., Messina A., Abberton K., Morrison W., Thompson E.W. (2007). Host rather than graft origin of Matrigel-induced adipose tissue in the murine tissue-engineering chamber. Tissue Eng..

[38] Wu L., Wang T., Ge Y., Cai X., Wang J., Lin Y. (2012). Secreted factors from adipose tissue increase adipogenic differentiation of mesenchymal stem cells. Cell Prolif..

[39] Suga H., Eto H., Aoi N., Kato H., Araki J., Doi K., Higashino T., Yoshimura K. (2010). Adipose tissue remodeling under ischemia: Death of adipocytes and activation of stem/progenitor cells. Plast. Reconstr. Surg..

[40] Guilliams M., Thierry G.R., Bonnardel J., Bajenoff M. (2020). Establishment and maintenance of the macrophage niche. Immunity.

[41] Gautier E.L., Shay T., Miller J., Greter M., Jakubzick C., Ivanov S., Helft J., Chow A., Elpek K.G., Gordonov S. (2012). Immunological Genome Consortium. Gene-expression profiles and transcriptional regulatory pathways that underlie the identity and diversity of mouse tissue macrophages. Nat. Immunol..

[42] Gosselin D., Link V.M., Romanoski C.E., Fonseca G.J., Eichenfield D.Z., Spann N.J., Stender J.D., Chun H.B., Garner H., Geissmann F. (2014). Environment drives selection and function of enhancers controlling tissue-specific macrophage identities. Cell.

[43] Lavin Y., Winter D., Blecher-Gonen R., David E., Keren-Shaul H., Merad M., Jung S., Amit I. (2014). Tissue-resident macrophage enhancer landscapes are shaped by the local microenvironment. Cell.

[44] Okabe Y., Medzhitov R. (2016). Tissue biology perspective on macrophages. Nat. Immunol..

