# Supplementary material for: Difference in M2 Macrophage Infiltration Between Cell-Assisted Fat Graft and Biomaterial-Encapsulated Stem Cell-Assisted Fat Graft: An Experimental Animal Study
Source: Medicina (Kaunas). 2026 Aug 6;62(8):1516. doi: 10.3390/medicina62081516 (PMC13515874; doi:10.3390/medicina62081516)
Supplement: Supplementary file 1 [file medicina-62-01516-s001.zip › medicina-4347592-supplementary.pdf]

(a)

CD163/CD68	SC	BEC	SC	BEC
n=1	0.5882	2.9660	1.5630	2.0710
2	0.5282	5.5520	0.7837	2.0162
3	0.3335	3.1698	0.7486	1.4471
4	2.5362	4.0706	0.5291	0.7480
5	7.1365	4.1323	1.1971	1.6451
6	7.4002	3.9024	1.7844	1.0020
Mean	3.087112	3.965506	1.100984	1.488242
Standard Deviation	3.3370	0.9156	0.4983	0.5346

(b)

	fat+ADSC 6W-1_R7-1	fat+ADSC 6W-1_R7-2	fat+ADSC 6W-1_R7-3	fat+ADSC 6W-3-R10	fat+ADSC 6W-3-R13-1	fat+ADSC 6W-3-R13-2
dapi	1790	1675	1849	10232	6177.556	7966.228
cy3	14770.530	9341.971	3570.125	35619.180	22361.184	45721.102
647	25111.080	17687.582	10705.658	14044.409	3133.366	6178.393
CD163/CD68	0.5882	0.5282	0.3335	2.5362	7.1365	7.4002
	1013 Bec-1 R2	1013 Bec-1 R14	Bec 6W-1 R20	Bec 6W-1 R21	Bec 6W-2 R22	Bec 6W-2 R23
dapi	1346.381	1044.62	839.544	1741.709	3236.374	2931.41
cy3	2468.443	6167.604	3600.225	4499.269	7669.99	7921.682
647	832.236	1110.88	1135.793	1105.318	1856.121	2029.964
CD163/CD68	2.9660	5.5520	3.1698	4.0706	4.1323	3.9024
	fat+ADSC 8w-1-R9-1	fat+ADSC 8w-1-R9-2	fat+ADSC 8w-1-R9-3	fat+ADSC 8w-1-R3	fat+ADSC 8w-1-R8-1	fat+ADSC 8w-1-R8-2
dapi	779.537	791.694	805.582	1751.792	4773.873	6383.885
cy3	3937.083	1957.493	1677.706	2404.124	4646.009	6655.753
647	2518.898	2497.697	2241.223	4543.879	3881.178	3729.868
CD163/CD68	1.5630	0.7837	0.7486	0.5291	1.1971	1.7844
	1013 Bec-2 R3-1	1013 Bec-2 R3-2	1013 Bec-2 R3-3	1013 Bec-2 R3-4	1013 Bec-2 R3-5	1013 Bec-2 R16
dapi	2640.855	1910.516	1320.491	1513.426	1444.119	1800.856
cy3(CD163)	2905.35	1426.64	2242.886	2963.515	2638.74	2042.654
647(CD68)	1402.85	707.585	1549.947	3961.938	1603.991	2038.512
CD163/CD68	2.0710	2.0162	1.4471	0.7480	1.6451	1.0020

(c) **T Test**

[DataSet3] D:\Evaluation of the Homing Potential of Adipose-Derived Stromal Cells after Distant Subcutaneous Injection\bead-Biomaterial Encapsulation on Stem Cell-Assisted Fat Grafts Increases M2 Macr...

Group Statistics

	SC/BEC Week	N	Mean	Standard Deviation	Standard Error Mean
VAR00002	SC-6W	6	3.087133	3.3370270	1.3623356
	BEC-6W	6	3.965517	0.9156387	0.3738079
VAR00004	SC-6W	6	1.100983	0.4983271	0.2034412
	BEC-6W	6	1.488233	0.5346089	0.2182532

Independent Samples Test

		Levene's Test for Equality of Variances		t-test for Equality of Means					95% CI of the Difference	
Variable	Assumption	F	Sig.	t	df	Sig. (2-tailed)	Mean Difference	Std. Error Difference	Lower	Upper
VAR00002	Equal variances assumed	12.880	.005	-.622	10	.548	-.8783833	1.4126891	-4.0260509	2.2692842
	Equal variances not assumed			-.622	5.749	.558	-.8783833	1.4126891	-4.3720797	2.6153130
VAR00004	Equal variances assumed	.004	.951	-1.298	10	.223	-.3872500	.2983668	-1.0520527	.2775527
	Equal variances not assumed			-1.298	9.951	.224	-.3872500	.2983668	-1.0524966	.2779966

Supplementary Figure S1. Quantification and statistical analysis of CD163+/CD68+ macrophage-related signals.(a) Relative CD163+/CD68+ macrophage-related signal ratios in the SC and BEC groups at postoperative weeks 6 and 8. The values were derived from image-based fluorescence signal analysis. Data are presented as mean ± SD. (b) Representative quantification table showing DAPI, Cy3, Alexa Fluor 647, CD163, and CD68 signal measurements used for the calculation of the relative CD163+/CD68+ signal ratio. (c) Independent-samples *t*-test results comparing the SC and BEC groups at weeks 6 and 8. Variability differed between the groups, with relatively larger variance observed in the SC group, particularly at week 6. The BEC group showed numerically higher relative CD163+/CD68+ signals at both time points; however, the differences were not statistically significant.
